# Ethyl (4aR*,7S*,8S*,8aS*)-1-oxo-7-phenyl-3,4,4a,7,8,8a-hexa­hydro-1*H*-isochromene-8-carboxyl­ate

**DOI:** 10.1107/S1600536810017009

**Published:** 2010-05-15

**Authors:** Xiu Qing Jiang, Jin-Long Wu

**Affiliations:** a Laboratory of Asymmetric Catalysis and Synthesis, Department of Chemistry, Zhejiang University, Hangzhou 310027, People’s Republic of China

## Abstract

In the title compound, C_18_H_20_O_4_, both the tetra­hydro­pyran­one ring and the cyclo­hexene ring adopt envelope conformations. The crystal packing is stabilized by weak inter­molecular C—H⋯O hydrogen bonding.

## Related literature

The title compound is a derivative of 1-oxo-hexa­hydro-1*H*-isochromene, which has been reported as a key inter­mediate towards the total syntheses of natural products such as eleutherobin and tetronothio­din, see: Kim *et al.* (2000 ▶); Jung *et al.* (2000 ▶); Page *et al.* (2003 ▶). For microwave-assisted intra­molecular Diels–Alder cyclo­addition, see: Wu *et al.* (2006 ▶, 2007 ▶); Wang *et al.* (2009 ▶).
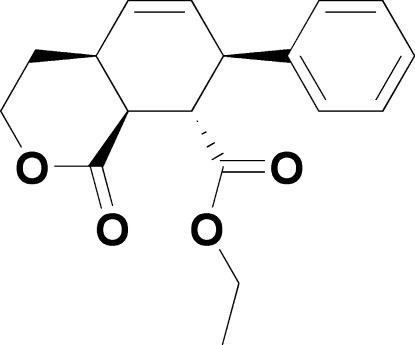


## Experimental

### 

#### Crystal data

C_18_H_20_O_4_
*M* *_r_* = 300.34
Orthorhombic, 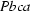

*a* = 15.5513 (12) Å
*b* = 9.9178 (7) Å
*c* = 21.1542 (17) Å
*V* = 3262.7 (4) Å^3^
*Z* = 8
Mo *K*α radiation
μ = 0.09 mm^−1^
*T* = 296 K
0.36 × 0.16 × 0.14 mm

#### Data collection

Rigaku R-AXIS RAPID IP diffractometer
26140 measured reflections
3200 independent reflections
1627 reflections with *I* > 2σ(*I*)
*R*_int_ = 0.075

#### Refinement

*R*[*F* ^2^ > 2σ(*F* ^2^)] = 0.069
*wR*(*F* ^2^) = 0.197
*S* = 1.00
3200 reflections
202 parameters
1 restraint
H-atom parameters constrained
Δρ_max_ = 0.35 e Å^−3^
Δρ_min_ = −0.28 e Å^−3^

### 

Data collection: *PROCESS-AUTO* (Rigaku, 1998 ▶); cell refinement: *PROCESS-AUTO*; data reduction: *CrystalStructure* (Rigaku/MSC, 2002 ▶); program(s) used to solve structure: *SHELXS97* (Sheldrick, 2008 ▶); program(s) used to refine structure: *SHELXL97* (Sheldrick, 2008 ▶); molecular graphics: *ORTEP-3 for Windows* (Farrugia, 1997 ▶); software used to prepare material for publication: *WinGX* (Farrugia, 1999 ▶).

## Supplementary Material

Crystal structure: contains datablocks I, global. DOI: 10.1107/S1600536810017009/xu2757sup1.cif

Structure factors: contains datablocks I. DOI: 10.1107/S1600536810017009/xu2757Isup2.hkl

Additional supplementary materials:  crystallographic information; 3D view; checkCIF report

## Figures and Tables

**Table 1:** Hydrogen-bond geometry (Å, °)

*D*—H⋯*A*	*D*—H	H⋯*A*	*D*⋯*A*	*D*—H⋯*A*
C1—H1⋯O4^i^	0.98	2.45	3.401 (4)	164
C5—H5*A*⋯O4^i^	0.97	2.48	3.412 (4)	161
C7—H7⋯O1^ii^	0.93	2.56	3.468 (5)	165
C13—H13⋯O2^iii^	0.93	2.59	3.356 (6)	140

Symmetry codes: (i) 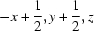; (ii) 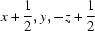; (iii) 
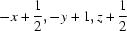
.

**Fig. 1.**
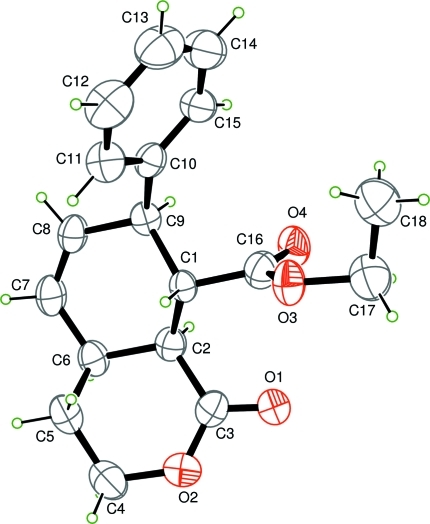
The molecular structure of the title compound with the atom numbering scheme. Displacement ellipsoids are drawn at 40% probability level. H atoms are presented as a small spheres of arbitrary radius.

## supplementary crystallographic information

### Comment

The title compound, C_18_H_20_O_4_, refers to the derivative of 1-oxo- hexahydro-1*H*-isochromenes, which has been reported as a key intermediate towards total syntheses of natural products such as eleutherobin (Kim *et al.*, 2000; Jung *et al.*, 2000) and tetronothiodin (Page *et al.*, 2003). The title compound has recently been obtained during microwave-assisted intramolecular Diels-Alder cycloaddition along with a minor diastereomer with a 74:26 diastereomeric ratio (Wu *et al.*, 2006, 2007; Wang *et al.*, 2009). The compound has four stereogenic centers but crystallizes as a racemate as indicated by the centrosymmetric space group. We report here its crystal structure.

In the molecular structure of the title compound (Fig. 1), there are one pyranone ring and one cyclohexene ring. Both of the two rings C1-C2/C6-C9 and C2-C3/O2/C4-C6 adopt envelope conformation. The crystal packing is stabilized by weak non-classical intermolecular C—H···O hydrogen bonds (Table 1).

### Experimental

A 10 mL pressured process vial was charged ethyl (3*E*,5*E*)-6-phenylhexa-3,5-dien-1-yl fumarate (172.0 mg, 0.57 mmol) followed by adding MeCN (4 mL). The loaded vial was then sealed with a cap containing a silicon septum and put into the cavity of a technical microwave reactor with the temperature measured by an IR sensor. After heating at 453 K for 1 h, the reaction mixture was concentrated under reduced pressure. The residue was purified by flash column chromatography (silica gel, 20% EtOAc in petroleum ether) to furnish the title compound (95.0 mg, 52%), along with a minor diastereomer (32.0 mg, 19%), as colorless needles. mp 392-394 K (EtOAc-hexane). Single crystals, as a racemate, suitable for X-ray diffraction of the title compound were grown at ambient temperature in the mixed solvent of ethyl acetate and hexane (v:v = 10:1).

### Refinement

H atoms were placed in calculated positions with C—H = 0.93-0.98 Å, and refined in riding model with U_iso_(H) = 1.5U_eq_(C) for methyl and 1.2U_eq_(C) for the others.

### Crystal data

**Table:**

C_18_H_20_O_4_	*F*(000) = 1280
*M**_r_* = 300.34	*D*_x_ = 1.223 Mg m^−^^3^
Orthorhombic, *P**b**c**a*	Mo *K*α radiation, λ = 0.71073 Å
Hall symbol: -P 2ac 2ab	Cell parameters from 12675 reflections
*a* = 15.5513 (12) Å	θ = 3.1–27.4°
*b* = 9.9178 (7) Å	µ = 0.09 mm^−^^1^
*c* = 21.1542 (17) Å	*T* = 296 K
*V* = 3262.7 (4) Å^3^	Needle, colorless
*Z* = 8	0.36 × 0.16 × 0.14 mm

### Data collection

**Table:**

Rigaku R-AXIS RAPID IP diffractometer	1627 reflections with *I* > 2σ(*I*)
Radiation source: rolling anode	*R*_int_ = 0.075
graphite	θ_max_ = 26.0°, θ_min_ = 3.1°
Detector resolution: 10.00 pixels mm^-1^	*h* = −19→19
ω scans	*k* = −11→12
26140 measured reflections	*l* = −26→26
3200 independent reflections	

### Refinement

**Table:**

Refinement on *F*^2^	Secondary atom site location: difference Fourier map
Least-squares matrix: full	Hydrogen site location: inferred from neighbouring sites
*R*[*F*^2^ > 2σ(*F*^2^)] = 0.069	H-atom parameters constrained
*wR*(*F*^2^) = 0.197	*w* = 1/[σ^2^(*F*_o_^2^) + (0.0698*P*)^2^ + 1.9409*P*] where *P* = (*F*_o_^2^ + 2*F*_c_^2^)/3
*S* = 1.00	(Δ/σ)_max_ < 0.001
3200 reflections	Δρ_max_ = 0.35 e Å^−^^3^
202 parameters	Δρ_min_ = −0.28 e Å^−^^3^
1 restraint	Extinction correction: *SHELXL97* (Sheldrick, 2008), Fc^*^=kFc[1+0.001xFc^2^λ^3^/sin(2θ)]^-1/4^
Primary atom site location: structure-invariant direct methods	Extinction coefficient: 0.0067 (12)

### Special details

**Table:**

Geometry. All esds (except the esd in the dihedral angle between two l.s. planes) are estimated using the full covariance matrix. The cell esds are taken into account individually in the estimation of esds in distances, angles and torsion angles; correlations between esds in cell parameters are only used when they are defined by crystal symmetry. An approximate (isotropic) treatment of cell esds is used for estimating esds involving l.s. planes.
Refinement. Refinement of *F*^2^ against ALL reflections. The weighted *R*-factor wR and goodness of fit *S* are based on *F*^2^, conventional *R*-factors *R* are based on *F*, with *F* set to zero for negative *F*^2^. The threshold expression of *F*^2^ > σ(*F*^2^) is used only for calculating *R*-factors(gt) etc. and is not relevant to the choice of reflections for refinement. *R*-factors based on *F*^2^ are statistically about twice as large as those based on *F*, and *R*- factors based on ALL data will be even larger.

### Fractional atomic coordinates and isotropic or equivalent isotropic displacement parameters (Å^2^)

**Table:**

	*x*	*y*	*z*	*U*_iso_*/*U*_eq_	
O3	0.16345 (14)	0.3940 (2)	0.39009 (15)	0.1040 (10)	
C17	0.0706 (3)	0.3651 (6)	0.3998 (3)	0.146 (2)	
H17A	0.0524	0.2860	0.3764	0.175*	
H17B	0.0350	0.4414	0.3880	0.175*	
C18	0.0687 (4)	0.3407 (8)	0.4701 (3)	0.205 (3)	
H18A	0.0993	0.2591	0.4796	0.308*	
H18B	0.0102	0.3325	0.4839	0.308*	
H18C	0.0955	0.4150	0.4915	0.308*	
O4	0.18555 (14)	0.1796 (2)	0.36346 (12)	0.0848 (8)	
C1	0.30154 (17)	0.3380 (3)	0.35293 (14)	0.0588 (8)	
H1	0.3083	0.4330	0.3645	0.071*	
C9	0.36348 (18)	0.2519 (3)	0.39280 (14)	0.0642 (8)	
H9	0.3484	0.1571	0.3860	0.077*	
C16	0.21088 (19)	0.2931 (3)	0.36799 (15)	0.0660 (8)	
C10	0.35139 (19)	0.2824 (3)	0.46291 (15)	0.0680 (8)	
C8	0.4540 (2)	0.2708 (3)	0.37126 (18)	0.0765 (9)	
H8	0.4978	0.2486	0.3993	0.092*	
O2	0.27531 (18)	0.5007 (3)	0.20646 (13)	0.1045 (9)	
C2	0.32050 (19)	0.3207 (3)	0.28126 (15)	0.0704 (9)	
H2	0.3127	0.2249	0.2714	0.084*	
C5	0.4213 (2)	0.5048 (4)	0.24889 (18)	0.0856 (10)	
H5A	0.4050	0.5586	0.2853	0.103*	
H5B	0.4803	0.5264	0.2381	0.103*	
C7	0.4757 (2)	0.3170 (4)	0.31524 (19)	0.0837 (11)	
H7	0.5340	0.3258	0.3065	0.100*	
C6	0.4140 (2)	0.3561 (3)	0.26494 (16)	0.0730 (9)	
H6	0.4293	0.3053	0.2268	0.088*	
C15	0.3062 (2)	0.1947 (4)	0.50117 (18)	0.0838 (10)	
H15	0.2835	0.1159	0.4842	0.101*	
C3	0.2542 (3)	0.3978 (4)	0.24313 (18)	0.0886 (11)	
O1	0.17953 (19)	0.3714 (4)	0.24613 (17)	0.1447 (14)	
C11	0.3852 (2)	0.3990 (4)	0.48850 (19)	0.0886 (11)	
H11	0.4150	0.4592	0.4629	0.106*	
C14	0.2948 (3)	0.2231 (6)	0.5638 (2)	0.1138 (14)	
H14	0.2636	0.1641	0.5891	0.137*	
C13	0.3294 (4)	0.3388 (7)	0.5899 (2)	0.1200 (16)	
H13	0.3222	0.3572	0.6326	0.144*	
C4	0.3635 (3)	0.5372 (5)	0.1941 (2)	0.1098 (14)	
H4A	0.3666	0.6330	0.1853	0.132*	
H4B	0.3835	0.4893	0.1570	0.132*	
C12	0.3746 (3)	0.4264 (5)	0.5521 (2)	0.1144 (15)	
H12	0.3982	0.5043	0.5693	0.137*	

### Atomic displacement parameters (Å^2^)

**Table:**

	*U*^11^	*U*^22^	*U*^33^	*U*^12^	*U*^13^	*U*^23^
O3	0.0557 (13)	0.0768 (15)	0.179 (3)	−0.0037 (11)	0.0323 (15)	−0.0228 (16)
C17	0.095 (3)	0.127 (4)	0.216 (7)	−0.015 (3)	0.045 (4)	−0.020 (4)
C18	0.146 (6)	0.258 (8)	0.211 (8)	−0.069 (5)	0.052 (5)	0.007 (7)
O4	0.0728 (15)	0.0589 (13)	0.123 (2)	−0.0162 (11)	−0.0052 (13)	−0.0008 (12)
C1	0.0494 (15)	0.0527 (15)	0.074 (2)	−0.0019 (13)	0.0044 (14)	−0.0056 (14)
C9	0.0597 (17)	0.0526 (15)	0.080 (2)	0.0045 (14)	0.0024 (16)	−0.0035 (15)
C16	0.0563 (17)	0.0572 (17)	0.084 (2)	−0.0022 (14)	−0.0007 (16)	0.0004 (16)
C10	0.0599 (18)	0.0660 (19)	0.078 (2)	0.0079 (15)	−0.0042 (16)	−0.0010 (17)
C8	0.0546 (18)	0.079 (2)	0.096 (3)	0.0106 (16)	0.0022 (18)	−0.0019 (19)
O2	0.095 (2)	0.116 (2)	0.103 (2)	−0.0024 (16)	−0.0076 (15)	0.0319 (17)
C2	0.068 (2)	0.0661 (18)	0.077 (2)	−0.0033 (16)	0.0021 (16)	−0.0063 (16)
C5	0.077 (2)	0.083 (2)	0.096 (3)	−0.0076 (19)	0.018 (2)	−0.002 (2)
C7	0.0534 (19)	0.091 (2)	0.106 (3)	0.0064 (17)	0.0163 (19)	−0.010 (2)
C6	0.068 (2)	0.074 (2)	0.077 (2)	0.0017 (16)	0.0155 (17)	−0.0103 (17)
C15	0.081 (2)	0.092 (2)	0.078 (2)	0.003 (2)	0.0015 (19)	0.011 (2)
C3	0.078 (2)	0.106 (3)	0.083 (2)	−0.007 (2)	−0.010 (2)	0.010 (2)
O1	0.0751 (19)	0.195 (3)	0.164 (3)	−0.024 (2)	−0.0326 (19)	0.072 (3)
C11	0.096 (3)	0.078 (2)	0.092 (3)	−0.001 (2)	−0.003 (2)	−0.015 (2)
C14	0.111 (3)	0.133 (4)	0.098 (4)	0.009 (3)	0.005 (3)	0.022 (3)
C13	0.127 (4)	0.155 (5)	0.078 (3)	0.037 (4)	0.003 (3)	−0.012 (3)
C4	0.099 (3)	0.111 (3)	0.119 (3)	−0.008 (3)	0.014 (3)	0.028 (3)
C12	0.128 (4)	0.109 (3)	0.106 (4)	0.014 (3)	−0.015 (3)	−0.031 (3)

### Geometric parameters (Å, °)

**Table:**

O3—C16	1.327 (4)	C2—C3	1.516 (5)
O3—C17	1.486 (5)	C2—C6	1.535 (4)
C17—C18	1.507 (8)	C2—H2	0.9800
C17—H17A	0.9700	C5—C4	1.501 (5)
C17—H17B	0.9700	C5—C6	1.518 (5)
C18—H18A	0.9600	C5—H5A	0.9700
C18—H18B	0.9600	C5—H5B	0.9700
C18—H18C	0.9600	C7—C6	1.485 (5)
O4—C16	1.197 (3)	C7—H7	0.9300
C1—C16	1.512 (4)	C6—H6	0.9800
C1—C9	1.539 (4)	C15—C14	1.367 (6)
C1—C2	1.554 (4)	C15—H15	0.9300
C1—H1	0.9800	C3—O1	1.192 (4)
C9—C8	1.491 (4)	C11—C12	1.382 (6)
C9—C10	1.525 (4)	C11—H11	0.9300
C9—H9	0.9800	C14—C13	1.382 (7)
C10—C15	1.379 (5)	C14—H14	0.9300
C10—C11	1.381 (4)	C13—C12	1.374 (6)
C8—C7	1.314 (4)	C13—H13	0.9300
C8—H8	0.9300	C4—H4A	0.9700
O2—C3	1.323 (4)	C4—H4B	0.9700
O2—C4	1.442 (5)	C12—H12	0.9300
			
C16—O3—C17	116.3 (3)	C1—C2—H2	106.9
O3—C17—C18	100.7 (4)	C4—C5—C6	109.6 (3)
O3—C17—H17A	111.6	C4—C5—H5A	109.7
C18—C17—H17A	111.6	C6—C5—H5A	109.7
O3—C17—H17B	111.6	C4—C5—H5B	109.7
C18—C17—H17B	111.6	C6—C5—H5B	109.7
H17A—C17—H17B	109.4	H5A—C5—H5B	108.2
C17—C18—H18A	109.5	C8—C7—C6	124.8 (3)
C17—C18—H18B	109.5	C8—C7—H7	117.6
H18A—C18—H18B	109.5	C6—C7—H7	117.6
C17—C18—H18C	109.5	C7—C6—C5	111.5 (3)
H18A—C18—H18C	109.5	C7—C6—C2	113.0 (3)
H18B—C18—H18C	109.5	C5—C6—C2	110.1 (3)
C16—C1—C9	107.8 (2)	C7—C6—H6	107.3
C16—C1—C2	110.5 (2)	C5—C6—H6	107.3
C9—C1—C2	110.8 (2)	C2—C6—H6	107.3
C16—C1—H1	109.3	C14—C15—C10	120.4 (4)
C9—C1—H1	109.3	C14—C15—H15	119.8
C2—C1—H1	109.3	C10—C15—H15	119.8
C8—C9—C10	112.9 (3)	O1—C3—O2	116.3 (4)
C8—C9—C1	110.7 (3)	O1—C3—C2	121.5 (4)
C10—C9—C1	110.2 (2)	O2—C3—C2	122.2 (3)
C8—C9—H9	107.6	C10—C11—C12	120.0 (4)
C10—C9—H9	107.6	C10—C11—H11	120.0
C1—C9—H9	107.6	C12—C11—H11	120.0
O4—C16—O3	123.6 (3)	C15—C14—C13	120.5 (5)
O4—C16—C1	124.5 (3)	C15—C14—H14	119.8
O3—C16—C1	111.8 (2)	C13—C14—H14	119.8
C15—C10—C11	119.4 (3)	C12—C13—C14	119.5 (4)
C15—C10—C9	120.6 (3)	C12—C13—H13	120.3
C11—C10—C9	120.0 (3)	C14—C13—H13	120.3
C7—C8—C9	124.2 (3)	O2—C4—C5	112.1 (3)
C7—C8—H8	117.9	O2—C4—H4A	109.2
C9—C8—H8	117.9	C5—C4—H4A	109.2
C3—O2—C4	122.4 (3)	O2—C4—H4B	109.2
C3—C2—C6	114.1 (3)	C5—C4—H4B	109.2
C3—C2—C1	109.5 (3)	H4A—C4—H4B	107.9
C6—C2—C1	112.0 (2)	C13—C12—C11	120.2 (4)
C3—C2—H2	106.9	C13—C12—H12	119.9
C6—C2—H2	106.9	C11—C12—H12	119.9
			
C16—O3—C17—C18	100.1 (5)	C8—C7—C6—C2	−7.3 (5)
C16—C1—C9—C8	168.7 (2)	C4—C5—C6—C7	175.1 (3)
C2—C1—C9—C8	47.7 (3)	C4—C5—C6—C2	−58.7 (4)
C16—C1—C9—C10	−65.7 (3)	C3—C2—C6—C7	160.7 (3)
C2—C1—C9—C10	173.3 (2)	C1—C2—C6—C7	35.6 (4)
C17—O3—C16—O4	−9.7 (6)	C3—C2—C6—C5	35.4 (4)
C17—O3—C16—C1	173.5 (3)	C1—C2—C6—C5	−89.8 (3)
C9—C1—C16—O4	−55.5 (4)	C11—C10—C15—C14	0.1 (5)
C2—C1—C16—O4	65.7 (4)	C9—C10—C15—C14	−179.3 (3)
C9—C1—C16—O3	121.3 (3)	C4—O2—C3—O1	−174.7 (4)
C2—C1—C16—O3	−117.5 (3)	C4—O2—C3—C2	7.5 (6)
C8—C9—C10—C15	−133.0 (3)	C6—C2—C3—O1	172.4 (4)
C1—C9—C10—C15	102.6 (3)	C1—C2—C3—O1	−61.2 (5)
C8—C9—C10—C11	47.5 (4)	C6—C2—C3—O2	−9.9 (5)
C1—C9—C10—C11	−76.9 (4)	C1—C2—C3—O2	116.5 (4)
C10—C9—C8—C7	−144.6 (3)	C15—C10—C11—C12	0.9 (5)
C1—C9—C8—C7	−20.5 (4)	C9—C10—C11—C12	−179.7 (3)
C16—C1—C2—C3	56.2 (3)	C10—C15—C14—C13	−1.0 (6)
C9—C1—C2—C3	175.6 (3)	C15—C14—C13—C12	0.8 (7)
C16—C1—C2—C6	−176.1 (2)	C3—O2—C4—C5	−31.0 (5)
C9—C1—C2—C6	−56.7 (3)	C6—C5—C4—O2	56.5 (4)
C9—C8—C7—C6	−0.5 (6)	C14—C13—C12—C11	0.2 (7)
C8—C7—C6—C5	117.3 (4)	C10—C11—C12—C13	−1.0 (6)

### Hydrogen-bond geometry (Å, °)

**Table:**

*D*—H···*A*	*D*—H	H···*A*	*D*···*A*	*D*—H···*A*
C1—H1···O4^i^	0.98	2.45	3.401 (4)	164
C5—H5A···O4^i^	0.97	2.48	3.412 (4)	161
C7—H7···O1^ii^	0.93	2.56	3.468 (5)	165
C13—H13···O2^iii^	0.93	2.59	3.356 (6)	140

Symmetry codes: (i) −*x*+1/2, *y*+1/2, *z*; (ii) *x*+1/2, *y*, −*z*+1/2; (iii) −*x*+1/2, −*y*+1, *z*+1/2.
